# How to Achieve a Healthier and More Sustainable Europe by 2040 According to the Public? Results of a Five-Country Questionnaire Survey

**DOI:** 10.3390/ijerph17176071

**Published:** 2020-08-20

**Authors:** Iva Zvěřinová, Vojtěch Máca, Milan Ščasný, Rosa Strube, Sibila Marques, Diana Dubová, Martin Kryl, Daniela Craveiro, Timothy Taylor, Aline Chiabai, Silvestre García de Jalón

**Affiliations:** 1 Environment Centre, Charles University, 162 00 Prague, Czech Republic; vojtech.maca@czp.cuni.cz (V.M.); milan.scasny@czp.cuni.cz (M.Š.); diana.dubova@czp.cuni.cz (D.D.); martin.kryl@czp.cuni.cz (M.K.); 2 Collaborating Centre on Sustainable Consumption and Production, 42107 Wuppertal, Germany; rosa.strube@scp-centre.org; 3 Instituto Universitário de Lisboa (ISCTE-IUL), CIS-IUL, 1649-026 Lisboa, Portugal; Sibila.Marques@iscte-iul.pt (S.M.); daniela.craveiro@gmail.com (D.C.); 4 European Centre for Environment and Human Health, University of Exeter Medical School, Truro TR1 3HD, UK; timothy.j.taylor@exeter.ac.uk; 5 Basque Centre for Climate Change, Biscaya, 48004 Pais Vasco, Spain; aline.chiabai@bc3research.org (A.C.); silvestre.garciadejalon@bc3research.org (S.G.d.J.)

**Keywords:** future scenarios, public health, sustainability, equity, green spaces, active mobility, housing, food consumption, values, public acceptability, policy support

## Abstract

The aim of this paper is to understand public preferences for several future scenarios of achieving a healthier, more equitable and sustainable Europe, which differ in the way the society is organized (individualistically vs. collectively) and in the driving sector (public vs. private). To achieve this aim, we conducted a questionnaire survey using representative samples for five European countries in 2018. About three thousand respondents chose among the four scenarios presented within four different contexts (green spaces, active mobility, energy-efficient housing, food consumption) or none of them. A majority of people in the five European countries were ready to accept one of the scenarios. We found significant differences in preferences according to socioeconomic backgrounds and values of respondents. People above 35 years old, those who were less educated, and those in the lowest household income tertile were less supportive of all scenarios. The heterogeneity in preferences associated with differences in socioeconomic backgrounds was larger for the scenario in which society is organized individualistically and driven by the private sector. Smaller distinctions were found in case of the scenario in which society is organized collectively and is driven by the public sector. Departing from social psychological theories, we examine the role of altruistic, biospheric, egoistic, hedonic, and security values. People with stronger biospheric values were more likely to accept scenarios, particularly those which are driven by the public sector and where there is more collective organisation. Those with a more egoistic value orientation were more likely to have higher preferences for scenarios where the private sector had a dominant role. The policy implications, in terms of the selection and framing of policy measures to enhance public support, are discussed.

## 1. Introduction

### 1.1. Motivation and Research Questions

Current transport, food and energy production and consumption in Europe all need to change significantly to improve health of people, health equity and the environment [1,2,3,4,5,6,7,8,9]. These changes constitute an integral part of the Sustainable Development Goals set by the United Nations [10]. To achieve such positive changes and identify the needed actions four scenarios were created within the INHERIT project (www.inherit.eu). These scenarios were designed to encourage triple win solutions (healthier, more sustainable lifestyles, as well as greater health equity). They describe what green spaces, active mobility, energy efficient housing or consumption of food and beverages may look like in Europe in the year 2040. However, they differ in the way the society is organized (individualistically versus collectively) and in the driving sector (public versus private) [11].

To successfully implement actions and policies included in the scenarios it would be key to gain public acceptability or support [12]. This study assesses public preferences in five European countries for these four scenarios in order to provide a better understanding of three main questions: (i) What scenarios are the most preferred in various domains of living and countries? (ii) Are there socioeconomic distinctions in the public acceptability of the scenarios? (iii) What value orientations would explain public acceptability of the healthier and more environmentally friendly scenarios?

The research questions stem from previous literature (Section 1.2) and results of qualitative focus group research on these scenarios—giving an in depth perspective on the preferences of people in certain income groups for the INHERIT scenarios in selected countries [13,14]. There is need to extend this work by using a quantitative approach—so see if similar findings to those in the focus groups hold for wider samples. In this article, we analyse data from a questionnaire survey we conducted in five European countries in 2018. In the questionnaire, respondents were asked to choose among the four scenarios of how green spaces, active mobility, energy efficient housing and food consumption might look like by the year 2040.

### 1.2. Literature Review and Hypotheses

Several previous studies have developed transport and diet scenarios aimed at increasing health or mitigation of climate change [15,16]. Other studies proposed energy scenarios for decarbonisation that included large-scale deployment of renewable energy, energy efficiency improvements, carbon capture and storage, and nuclear energy [17,18]. A diversity of approaches, methods, policy scenarios, evaluation metrics and assumptions prevails. We found a consensus among the studies that change is needed, for example that reducing motor vehicle transport and meat consumption would lead to both better health and reduced emissions [15,16,17,18].

However, most of the studies on the scenarios of Europe in 2030–2050 concentrated only on one goal (such as the mitigation of climate change) and one domain of development, typically from an expert perspective. Policies cannot succeed if they are not also supported by citizens’ actions and voting practices [19], which are not enacted in isolation. Therefore, several studies examined public acceptability of policies, such as climate change or energy polices [12,20,21,22,23]. Our study builds on this literature and examines preferences of lay people for healthier and more environmentally friendly policies and behaviours that are described in the scenarios of development of four domains (green spaces, active mobility, energy efficient housing, and food consumption). The four domains are experimental treatments that enable us to analyse differences in choices of scenarios under different contexts.

Only a few studies included the views of lay people on scenarios and explored their visions of the future from several aspects [24]. Young Europeans created their future life in 2040 in relation to consequences for land use in a questionnaire survey. Overall, they desired a change toward sustainability, particularly of lifestyles (local and environmentally friendly food production, eating less meat, having access to green space and the ability to go to work by bike). However, they preferred owning family houses with gardens, international travel and extensive food production at the same time. This study used crowdsourcing as a sampling method, which led to a geographic bias, and an over-representation of respondents with a higher education (81%), while the other sample characteristics seemed reasonably representative [24]. 

Furthermore, there are few recent public opinion polls representative of the EU countries that asked questions related to the future of the environment, health, and health equity in Europe [20,25,26]. Regarding environment protection, Europeans think that the European Union should favour the preservation of natural resources (41%), the further deployment of renewable energies (39%) and an increase in recycling and waste sorting (38%). The opinions differ according to countries and sociodemographic variables. Youth (15–24 years old) in comparison to people older than 55 years, higher educated respondents (who had completed education aged 20 and over), managers, and people who position themselves in the upper class (in comparison to the working class) are more likely to give priority to further development of renewable energies [25]. 

The majority of Europeans (78%) perceive that environmental problems have a direct impact on their daily life and their health [20]. When respondents are forced to select only three options that would describe the ideal future for the EU, Europeans favour ‘equal wages for the same job across the EU’ the most (38%). Around one third of Europeans perceive a minimum level of guaranteed healthcare in all EU countries as the ideal future for the EU. People who think they belong to the upper class are much more likely to choose this goal. A minimum level of guaranteed healthcare is prioritized over ‘increased use of renewable energies within the European Union’ (23%). However, these polls let people evaluate particular goals or measures and not coherent scenarios of future development. 

In this paper, we examine the preferences of lay people for the four scenarios, which differ in the way the society is organized (individualistically versus collectively) and in the driving sector (public versus private). We build on Schwartz’s Value Theory [27], which categorises people’s values based on whether they motivate people to focus on their own personal interests (“self-enhancement” values), or to transcend self-interest and accentuate collective interests (“self-transcendence” values). 

Value orientations, specifically egoistic, altruistic, and biospheric, were suggested as a theoretical basis of environmental concern and environment-related behaviours [28,29,30]. Several empirical studies have shown that a conflict between immediate individual gains and long-term collective interests is often a part of adopting more environmentally friendly behaviour. Thus, “self-transcendent” (i.e., altruistic or biospheric) versus “self-enhancement” (i.e., egoistic) value dimensions can explain different types of environmental beliefs and behaviours, including support for environmental policies (for literature overview see [30,31]). In general, people who hold strong biospheric and, to a lesser extent, altruistic values tend to evaluate more positively and are more likely to adopt sustainable energy behaviours, while people who hold strong egoistic and/or hedonic values are less likely do so [32]. The same tendency was found for support for pro-environmental policy measures [31]. Hedonic values make people emphasise pleasure and comfort, egoistic values motivate people to protect their personal resources (such as financial resources or status), altruistic values make people pay attention to the welfare of other people, and biospheric values motivate people to consider impacts on nature and the environment [33]. The motivational goal of security includes health, safety, harmony, and stability of society, of relationships, and of self. Security values belong primarily to “conservation” value dimension and are closer to “self-enhancement” value dimension [27]. 

Departing from Schwartz’s Value Theory [27] and the previous studies, we examine the role of altruistic, biospheric, egoistic, hedonic, and security values in acceptability of the scenarios. We hypothesise that acceptability of the scenarios will increase with strong biospheric (H1a), altruistic (H1b), and security values (H1c), while it will decrease with strong egoistic (H1d) and/or hedonic values (H1e). Further, we hypothesise that people will be more likely to prefer the development of a collectively organised society driven by the public sector, when they hold “self-transcendent” values that is altruistic (H2a) and biospheric (H2b). In contrast, people will tend to favour individualistically organised society driven by the private sector, where they possess “self-enhancement” values that is egoistic (H2c) and hedonic (H2d).

Our approach overcomes the limits of the previous studies by: (i) asking people to make a choice of the best pathway among the four scenarios; (ii) analysing data representative of adult populations (18 to 65 years) of five European countries; (iii) explaining the potential support for the scenarios by values and socio-economic characteristics; (iv) examining preferences for future scenarios when they are presented within four different contexts defined by four domains of living.

## 2. Materials and Methods 

### 2.1. Data

Data analysed in this article come from a questionnaire online survey we conducted in the Czech Republic, Latvia, Portugal, Spain, and the United Kingdom in 2018. The five countries were selected based on their different political and socio-economic contexts for the purpose of comparison. We surveyed the inhabitants of these countries aged between 18 and 65. The country subsamples were selected using quota sampling from online access panels provided by private public opinion companies [34].

The dataset excluding speeders consists in a total of 10288 observations [34]. Due to survey time constraints, some respondents did not evaluate the scenarios. Furthermore, each respondent was asked about preferred scenario in only one domain—either green spaces, active mobility, energy efficient housing or the consumption of food and beverages. We assigned the evaluation of scenarios and the domain of the scenarios to respondents randomly. For this reason, we analysed a dataset with 3222 observations in this paper. The reduced sample proportions deviated from the few quotas set. Based on the population shares (see Supplementary Materials, Tables S1 and S2), we derived weights to make the new dataset representative of national populations aged 18 to 65 years with respect to gender, age, education, and region. We have used these weights for data analysis in this paper. 

Ethical approvals of the survey have been obtained from the Ethical Committee of Charles University Environment Centre, University of Exeter Medical School Research Ethics Committee, Ethics Committee of University of Alcalá, Ethics Committee of ISCTE—University Institute of Lisbon, and Riga Stradins University Ethical Committee. The questionnaire was prepared based on a pre-survey, which took the form of 27 one-on-one semi-structured interviews, intensive pretesting, and a pilot survey (for details see [34]). One part of the questionnaire related to the scenarios was developed based on findings from a qualitative survey, which included 15 focus groups in five European countries and explored citizens’ perceptions of the same triple win scenarios [11,14].

### 2.2. Brief Description of the Scenarios

**Figure 1:**
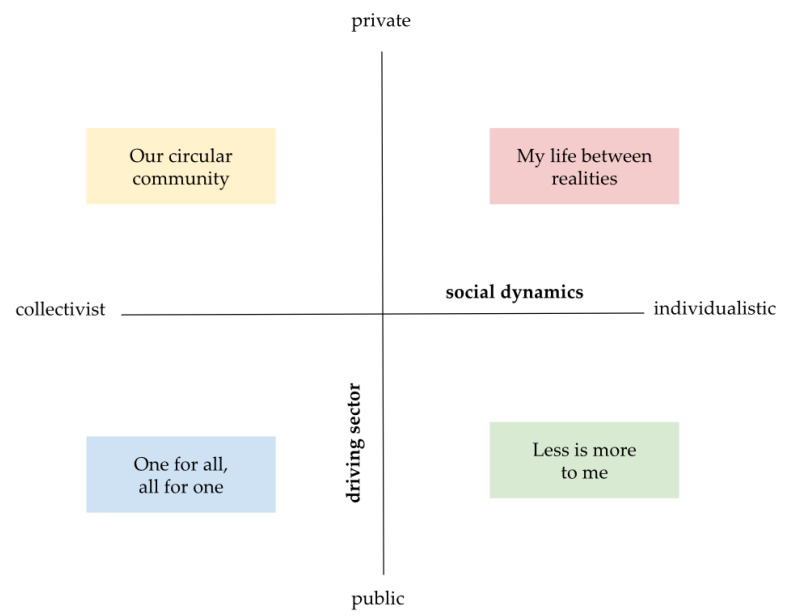
The main characteristics of the four future scenarios (adapted from [14]).

The aim of developing the scenarios was to inspire policy makers and other stakeholders [11]. The scenarios are simulations of possible future that envisions the new sustainable lifestyles and their links with societal, technological and policy developments [35]. They are visions, not predictions. Scenarios are a methodological tool that takes into account the available options and the likely consequences [36]. In this subchapter, we briefly describe the main characteristics of the scenarios (see Table 1 and Figure 1). The scenarios and the process of their development is described in detail in two studies [11,14]. The four scenarios differ in the way the society is organized (individualistically versus collectively) and in the driving sector (public versus private). 

**Figure A1:**
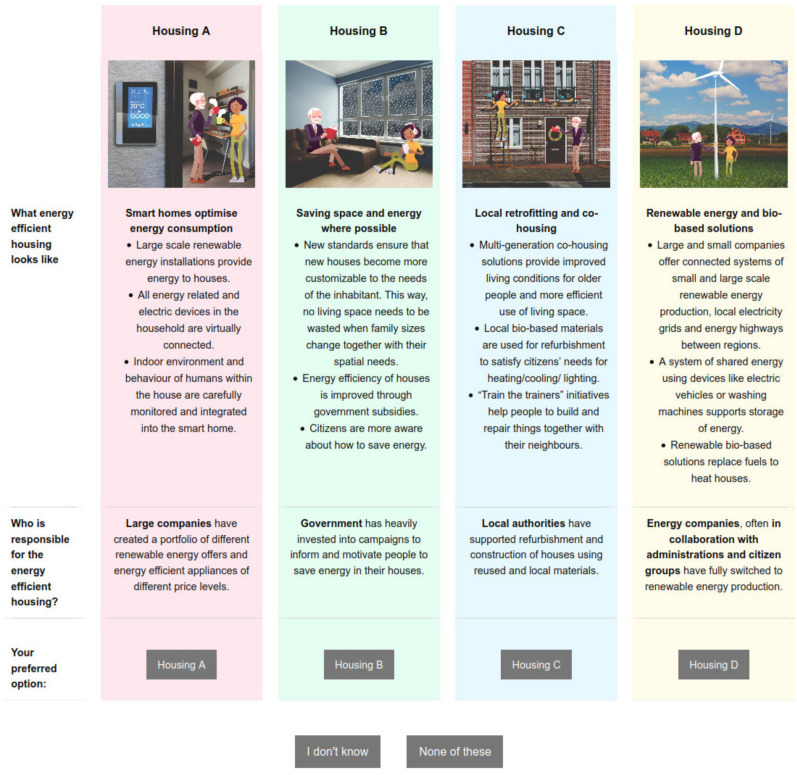
Example of a choice card—INHERIT scenarios, Energy efficient housing domain (Housing A corresponds to the “My life between realities” scenario, Housing B to the “Less is more to me” scenario, Housing C to the “One for all, all for one” scenario, and Housing D to the “Our circular community” scenario).

**Figure A2:**
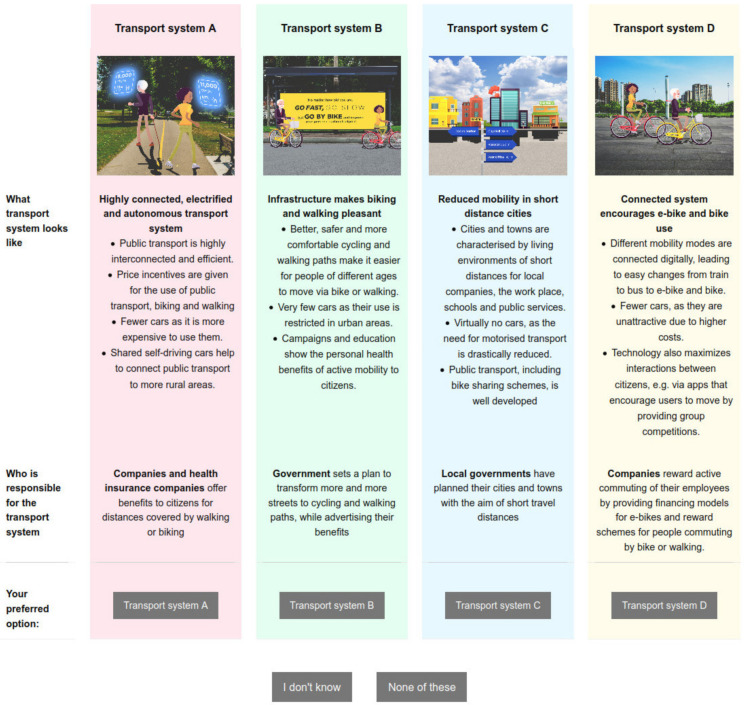
Example of a choice card—INHERIT scenarios, Active mobility domain (Transport system A corresponds to the “My life between realities” scenario, Transport system B to the “Less is more to me” scenario, Transport system C to the “One for all, all for one” scenario, and Transport system D to the “Our circular community” scenario).

**Figure A3:**
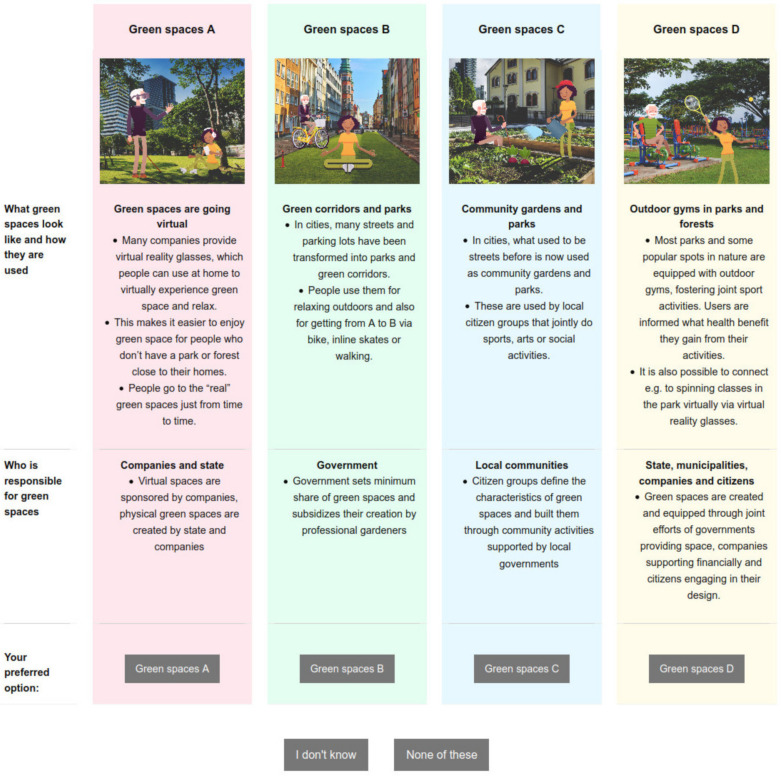
Example of a choice card—INHERIT scenarios, green spaces domain (Green space A corresponds to the “My life between realities” scenario, Green space B to the “Less is more to me” scenario, Green space C to the “One for all, all for one” scenario, and Green space D to the “Our circular community” scenario).

**Figure A4:**
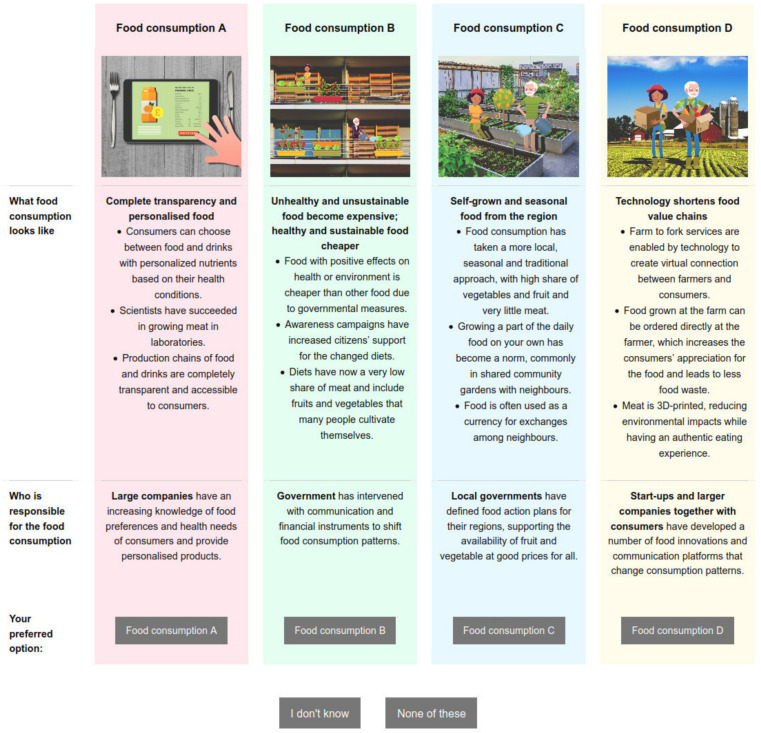
Example of a choice card—INHERIT scenarios, food consumption domain (Food consumption A corresponds to the “My life between realities” scenario, Food consumption B to the “Less is more to me” scenario, Food consumption C to the “One for all, all for one” scenario, and Food consumption D to the “Our circular community” scenario).

The scenarios’ focus is on four domains, namely green spaces, active mobility, energy efficient housing, and consumption of food and beverages. In the questionnaire survey, one domain was randomly assigned to each respondent. This allowed us to analyse the effects of different contexts of the choices. Respondents were prompted to imagine that they could choose between different governmental approaches that would influence what the given domain (green spaces, transport system, housing, or food consumption) in their country would look like by the year 2040. They were informed that all options should improve the state of domain (for example the share of green spaces should be larger than nowadays), which would have positive health and environmental impacts. Each respondent was then invited to indicate preferred scenario in only one domain. Respondents were asked to choose one of the four scenarios, none of them, or an ‘I don’t know’ option (see Figure A1, Figure A2, Figure A3 and Figure A4 in Appendix B for descriptions of the scenarios as presented to respondents). 

The choice card described the key features of the domain under each scenario and how responsibilities are shared between private and public sectors. The information provided about the scenario options included specific measures that would be undertaken by various actors to reach the health and environmental improvements. For example, in the case of green spaces in the scenario ‘Less is more to me’, the government sets the minimum share of green spaces and subsidizes their creation by professional gardeners. In contrast, citizen groups define the characteristics of green spaces and build them through community activities supported by local governments under the scenario ‘One for all, all for one’.

### 2.3. Values

**Figure 2:**
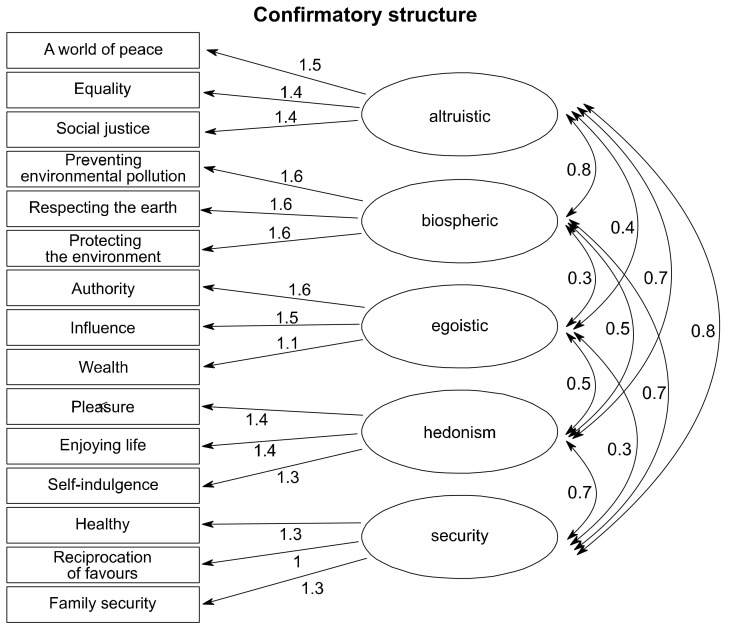
Factor loadings for value items and correlations between latent factors from confirmatory factor analysis.

Because we were particularly interested in the self-transcendence versus self-enhancement dimension [27], we selected values that belonged to these dimensions (altruistic and biospheric values versus egoistic and hedonic values). The security was chosen mostly because of the health value. People were asked to indicate on a 9-point scale ranging from 7 (‘of supreme importance’) to 0 (‘not important’) and −1 (‘opposed to my values’) how important each of the values is as a guiding principle in their life. The questionnaire included 16 items: 3 to measure the egoistic value orientation, 3 to measure the altruistic value orientation, and 3 to measure the biospheric value orientation, 3 to measure hedonism, and 4 to measure security [27,30]. The value items and reliabilities of value constructs are listed in Table A1 in Appendix A. The coefficient of reliability (Cronbach’s α) of all value constructs ranged from 0.73 to 0.91 exceeding 0.70, which is a recommended value [37]. Further, we carried out confirmatory factor analysis (CFA) in order to validate distinctions between the value orientations (latent factors) defined on theoretical grounds. Figure 2 depicts the path diagram of our CFA model including factor loadings for value items and correlations between latent factors. Approximate fit indexes of the CFA (Comparative Fit Index = 0.962; Tucker-Lewis Index = 0.950) indicate a good fit of the model. The root mean square error of approximation (RMSEA = 0.061) indicates a reasonable approximate fit, and is not far from value 0.05, which indicates a close approximate fit [38]. Therefore, we could create multi-item scales of egoistic, altruistic, and biospheric value orientation, hedonism, security. We computed mean scores for items that belong to the scales. We use these scales in the further analyses.

### 2.4. Socio-Economic Background

In order to identify the socio-economic background of respondents and to compare different population segments, we included several socio-demographic questions in the questionnaire. We elicited gender (male, female, other), age, the highest level of education, the approximate population size of the residence, and household monthly income from all sources after tax and compulsory deductions. For regression analysis, the variables age and the highest level of education have been simplified into three categories (age of 18–34 years, 35–49 years, and 50–65 years; primary and lower secondary, upper secondary, and tertiary education). Household income has been categorized according to terciles (1st tercile; 2nd tercile; 3rd tercile) and one dummy variable was created for missing income (I don’t know/no response). Municipality size enters in the regression analyses as a dummy variable for towns and cities with 5000 people or more. We also asked respondents whether they had chronic health problems (particularly cardiovascular disease, cancer, diabetes, food intolerance or allergy, stomach and other gastrointestinal diseases, any other chronic disease) or not.

### 2.5. Analyses

First, we provide descriptive statistics for support for the scenarios to find which of the four future scenarios is the most supported by inhabitants of the five EU countries (related statistical tests in Supplementary Materials, in Tables S4 and S5). 

Second, we conducted confirmatory factor analysis (CFA) in order to assess the fit between our data and a theoretically grounded model of relations between value orientations (latent factors) and their value items (observed indicator variables) [39]. The CFA was run using the R lavaan package [40]. Based on the CFA, we confirmed that egoistic, altruistic, and biospheric value orientation, hedonism, and security form distinct latent factors (Figure 2).

Third, we estimated multinomial logistic regressions to analyse relationships between socio-demographic characteristics, values, and domains on one side, and the choice of the four scenarios on the other side (in reference to no choice of the scenarios). The models are estimated for the dataset pooling the data for all five countries that control for the effects of countries using country dummy variables. Further, we depict predicted probabilities of scenario choices by biospheric and egoistic value orientation to help us to understand the results of the model.

All survey variables used in the regression analysis are described in Table S3 and Figure S1 in Supplementary Materials. We also estimated multinomial logit models with all possible combinations of reference categories that helps with interpretation (Table S6a–e in Supplementary Materials). In the paper, however, we show results of the model where the “I don’t know” and “None of these” form one reference category. The reason for this is that multinomial logit model with all six categories cannot be meaningfully interpreted because of a large share of empty cells (83%). This is because there is only a small number of respondents who selected “None of these” (N = 143, 4% from the whole sample). Moreover, the choices between “I don’t know” and “None of these” option are not systematically different based on binary logistic regression (the only significant difference is for age and Latvia; the details are available on the authors’ request). This model with all key explanatory variables included did not reduce −2LL statistic compared to the baseline model, implying that group of respondents who selected the two options seems to be rather homogenous. 

## 3. Results

### 3.1. What Scenarios Are the Most Preferred in Various Domains of Living and Countries? 

**Figure 3:**
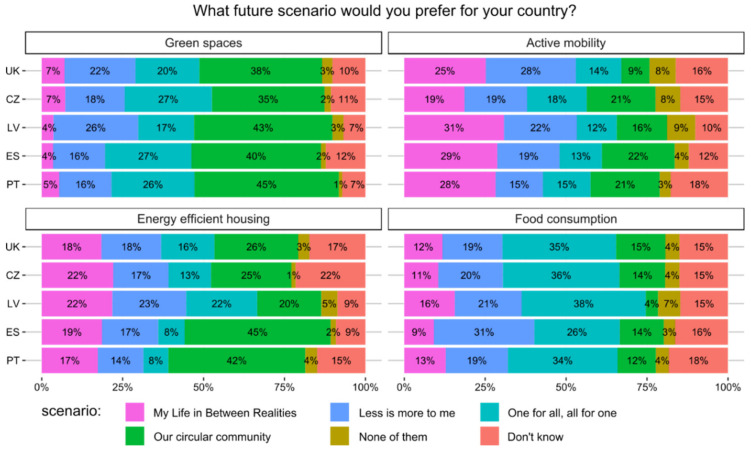
The most preferred scenarios in the domains of green spaces, active mobility, energy efficient housing, and food consumption (in percentages of respondents from the United Kingdom, Czech Republic, Latvia, Spain, and Portugal).

The preferences of inhabitants of the five European countries for four future scenarios in the domains of green spaces, active mobility, energy efficient housing or consumption of food are depicted in Figure 3. Based on statistical tests and multinomial logistic regressions, reported in the Supplementary Materials, in Tables S4–S6e, there are several significant differences among domains, while the differences across the five EU countries are much less pronounced.

In all domains, the majority of people (74% to 93%) preferred one of the healthier and more environmentally friendly scenarios. Only a minority of respondents (1% to 8%) selected none of them and 6% to 22% didn’t know. 

In the ‘Green spaces’ domain, the most commonly chosen was the ‘Our circular community’ scenario and at least preferred was the ‘My life between realities’ one in all analysed countries. People in all these countries tend to prefer real green spaces and spending leisure time outdoors over augmented reality. From 35% of Czechs to 45% of Latvians favoured the ‘Our circular community’ scenario, which suggested that most parks and some popular spots in nature would be equipped with outdoor gyms. Only a small share of people (4% to 6%) would like green space access to be virtual. There are no significant differences among countries for choices within green spaces domain.

In the ‘Active mobility’ domain, respondents would clearly like the transport system to become more interconnected with fewer cars. ‘My life between realities’ scenario was the most preferred in Spain (31%), Portugal (26%), and Latvia (30%). It was the second most preferred in the two remaining countries, but only by a statistically insignificant margin after ‘Less is more to me’ in the UK and ‘Our circular community’ in the Czech Republic. The ‘My life between realities’ scenario is based around the development of a highly connected, electrified and autonomous transport system, which includes highly interconnected and efficient public transport, price incentives for the use of public transport, biking and walking, fewer cars as it is more expensive to use them, and shared self-driving cars. The ‘Less is more to me’ scenario highlighted change in infrastructure that would make biking and walking pleasant and was supported by 28% of respondents from the UK. Compared to other countries, those living in the UK were far less likely to support ‘Our circular community’ for this domain, which emphasised development of a digitally connected transport system that encourages e-bike and bike.

In the ‘Energy efficient housing’ domain, many people see the future in renewable energy production and sharing of energy using devices. The most commonly chosen scenario in this domain was ‘Our circular community’—except in Latvia where it was significantly less preferred (where ‘My life between realities’ was the most preferred one). In ‘Our circular community’ scenario, large and small companies offer connected systems of small and large scale renewable energy production, local electricity grids and energy highways between regions. A system of shared energy using devices like electric vehicles or washing machines supports storage of energy. While the support for this scenario was clearly more prevalent in Spain (44%) and Portugal (41%), it was less dominant in the other countries (26% in the UK and 25% in the Czech Republic). 

In the ‘Food consumption’ domain, people would rely on the increasing trend of eating self-grown and seasonal food, more vegetables and fruits and low meat consumption. The ‘One for all, all for one’ scenario was the most selected in four survey countries in this domain. One third of Czech, British, and Portuguese respondents and even 40% of Latvians have chosen this scenario. In this scenario, food consumption follows a more local, seasonal and traditional approach (with high share of vegetables and fruit and very little meat), growing a part of the daily food has become a norm (commonly in shared community gardens with neighbours), and food is often used as a currency for exchange among neighbours. In Spain, the ‘Less is more to me’ (30%) and ‘One for all, all for one’ (28%) were the favourite scenarios. In all other countries, the ‘Less is more to me’ scenario was the second most popular (19% to 21% depending on the country). This scenario put emphasis on unhealthy and unsustainable food becoming more expensive and healthy and sustainable food cheaper. It was explicitly stated that to reach this goal the governments intervened with communication and financial instruments. This indicates a great acceptability of these instruments by 19% of Latvian respondents to 30% of Spanish respondents. ‘Our circular community’ was significantly less chosen in Latvia than in other countries.

### 3.2. Which Population Segments Are More Willing to Support the More Sustainable and Healthier Scenarios?

The results of multinomial logistic regression (Table 2) show that visions of the future differ mostly according to value orientation, socio-economic characteristics, and domain. There are only few significant differences among countries, which we have described in the preceding subchapter. 

Those aged over 35 years old are significantly less supportive of all future scenarios in comparison to the youngest age category. However, the strongest age divide is in preferences for the ‘My Life in Between Realities’ scenario, which highlights the spread of digital technologies and personalized services. The smallest but still significant difference between people under and above 35 years old is in the case of the scenario ‘One for all, all for one’, in which local authorities are the driving forces behind everyday living.

The tertiary and upper secondary educated are more prone to choose all future scenarios than those who are less educated. The strongest distinction between these education categories occurred in case of ‘My Life in Between Realities’ and ‘Less is more to me’. The only exception is scenario ‘One for all, all for one’, where the effect of tertiary education is not significant.

Income is positively associated with support for all four healthier, equitable and sustainable future scenarios. The largest income differences can be found in the choices of ‘My Life in Between Realities’, in which the society is organized individualistically and the private sector dominates over the public sector. People in the highest income tertile favour ‘My Life in Between Realities’ much more than those in the lowest income group. The ‘Our circular community’ scenario is more chosen both by people in the second and third tertiles. Not so large income distinctions can be seen in the case of ‘One for all, all for one’ scenario, in which society is organized collectively and public sector dominates over private sector. Respondents who did not provide information on their income, were more likely to choose none of the scenarios or the “I don’t know” option. The reason might be that these respondents did not like to reveal information to others trough the questionnaire or did not pay enough attention to answering the survey.

Size of residence a respondent lives and having a chronic health condition were not found to have significant effect on respondents’ choice of any future scenarios. We also found no significant differences in preferences of men and women.

The altruistic orientation, hedonism orientation and security orientation have no significant effect on the choice of scenarios. Thus, hypotheses H1b, H1c H1e H2a, and H2d can be rejected. Supporting the hypothesis H1a, the stronger biospheric values the higher the public acceptability of the scenarios.

**Figure 4:**
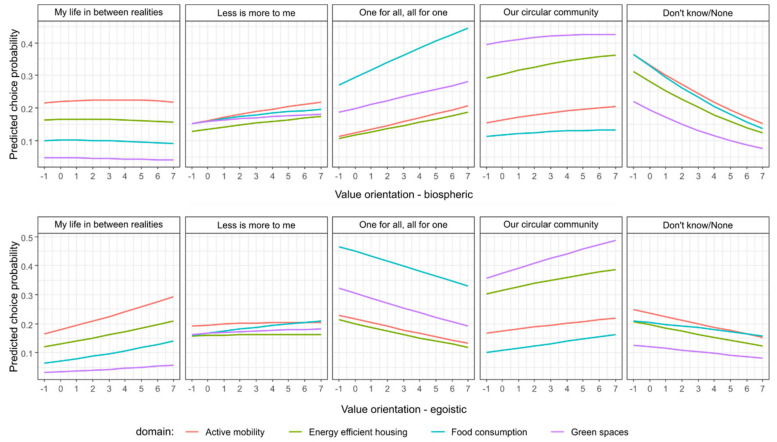
Predicted probabilities of scenario choices by biospheric and egoistic value orientation for domains of living (Predicted probabilities from the multinomial logit model presented in Table 2, fixed at country = CZ, household income = “2nd tercile”, municipality size = “town”, age = “35–49 years”, Education = “upper secondary”, gender = “Female”, Chronic disease = ”none”, means for altruistic, hedonic, and security values, and (alternatively) biospheric or egoistic value orientations).

The relationship between biospheric and egoistic value orientation and the scenario choices is illustrated by Figure 4. The higher the biospheric values the less likely it is that people would choose none of the scenarios or not know which one to choose, which provides further support to hypothesis H1a.

People with biospheric values particularly favour the public sector driven and collectivistic oriented scenario ‘One for all, all for one’, which is in support of H2b. The strongest effect of the biospheric values on the choice of ‘One for all, all for one’ scenario is in the case of food consumption. The more biospheric values people hold the slightly more likely they will be to accept the ‘Less is more to me’ and ‘Our circular community’ scenarios in all domains of living. Although the positive effect of biospheric values on preference for ‘My Life in Between Realities’ is significant (Table 2), Figure 4 shows that the actual effect size is very small or even non-existent. 

The higher egoistic orientation of people the more likely they choose a scenario with a dominant role of private sector in societal development, which are ‘My Life in Between Realities’ and ‘Our circular community’, supporting H2c. The effect of the egoistic values on preference for ‘My Life in Between Realities’ is most visible in the case of active mobility and least apparent in the case of green spaces. The more egoistic values people possess the less acceptable is the scenario ‘One for all, all for one’ for them.

## 4. Discussion

The majority of the population of five European countries (from 74% to 93%) would accept governmental approaches that would influence the development of four domains of living to improve health and the environment. The potential to accept the scenarios differs mostly according to the domain of living, value orientation, and socio-economic characteristics of respondents. There are only a few significant differences among countries.

### 4.1. What Scenarios Are the Most Preferred in Various Domains of Living and Countries?

Regarding food consumption, the scenario that highlights the local, seasonal and traditional approaches dominated the choices. It was supported by a third of Czech, British, and Portuguese respondents and even 40% of Latvians. This corresponds well to a ‘local’ food trend among consumers found in other studies [24,41,42,43]. The reason for this popularity is connected to perception of local food as environmentally friendlier, fresher and healthier than imported foods. Moreover, local food tends to be perceived as not as expensive as organic food [41]. The second most popular scenario would change the price of food, so unhealthy and unsustainable food would become more expensive and healthy and sustainable food cheaper. Use of financial policy instruments complemented by communication instruments would be acceptable for 30% of Spanish respondents and 20% of respondents from the other surveyed countries. 

People in all the countries surveyed tend to prefer real green spaces and spending leisure time outdoors over virtual reality. This corresponded to the qualitative results of focus groups [13,14]—where some noted the benefits for those who could not access green spaces, but felt they themselves would prefer the real world experience. The most favoured was the ‘Our circular community’ scenario (from 35% of Czechs to 45% of Latvians), which put the focus on most parks and some popular spots in nature being equipped with outdoor gyms. Few studies from non-European countries have shown that outdoor gyms mostly serve for adult and older adult groups. Outdoor gyms seem to be acceptable even for older adults according to an Australian study which showed that forty-two percent of park users above 50 years old had used an outdoor gym [44]. Outdoor gyms seem not only to be positively evaluated by users as pursuing health [45], but also to be used on a regular basis and thus raise the level of moderate to vigorous physical activity in parks [46]. Based on a natural experiment in urban public parks, building fitness zones seems to be cost-effective (10.5 cents/MET increase) and most effective in parks in densely populated areas with few other facilities [46]. However, evidence on the long-term effects of fitness zone installations on physical activity is missing. 

In the ‘Active Mobility’ domain, respondents would like transport system to become more interconnected with fewer cars. The ‘My life between realities’ scenario was the most preferred in Spain (31%), Portugal (26%), and Latvia (30%). The ‘My life between realities’ scenario aims at developing a highly connected, electrified and autonomous transport system, which includes highly interconnected and efficient public transport, price incentives for the use of public transport, biking and walking, fewer cars as it is more expensive to use them, and shared self-driving cars. High public acceptability of automated vehicles has been found in Eurobarometer [47] as well, albeit only in cases where the vehicles are supervised by a human operator in them (70%). Our results suggest that economic interventions can become publicly acceptable, when they are framed as part of a coherent scenario. This is important, as a recent review and cost-effectiveness study indicates that the rise of fuel excise taxation may lead to health benefits due to obesity reduction and an increase in physical activity [48]. Our ‘My life between realities’ scenario is designed to overcome distributional concerns of fuel excise taxation because it includes subsidies for public transport [49], biking and walking, and company and health insurance benefits for biking and walking. The earmarking of revenues for public transport and alternative means of transportation, and development of clean technologies might have led to larger public acceptability of this scenario. Such an effect of the earmarking of revenues has been shown in a Norwegian study [50]. 

In the ‘Energy efficient housing’ domain, many people see the future in renewable energy production and sharing of energy-using devices. The most commonly chosen scenario was ‘Our circular community’—except in the Latvian case where it was significantly less preferred (where ‘My life between realities’ was the most preferred one). In ‘Our circular community’ scenario, large and small companies offer connected systems of small and large scale renewable energy production, local electricity grids and energy highways between regions. A system of shared energy using devices like electric vehicles or washing machines supports the storage of energy. While the support for this scenario was clearly significantly prevailing in Spain (44%) and Portugal (41%), it was much less dominant in the other countries (26% in the UK, 25% in the Czech Republic, and 19% in Latvia). These country differences are consistent with Special Eurobarometer’s result [26] that almost all respondents from Portugal and Spain (98%) agreed with the statement “EU’s responsibility is to encourage more investment in renewable energy”, while 82% agreed in the Czech Republic, 86% in Latvia and 89% in the UK. The percentages of agreement from the Eurobarometer are much higher than the percentages of potential support from our survey. This is most likely due to different measurements and that the scenarios in our study do not only relate to renewable energy. While there was choice among different approaches in the development of housing (energy savings, smart homes or retrofitting) in our survey, the agreement with the general statement on renewable energy was the only factor considered in the Eurobarometer survey.

### 4.2. Are There Socioeconomic Distinctions in the Public Acceptability of the Scenarios?

Overall, people above 35 years old, primary and lower secondary educated, in the lowest household income tertile were less supportive of all the healthier and more environmentally friendly scenarios than the others. Several reviews have shown that less educated, lower income or people of lower occupational status tend to have unhealthier diets [51,52]. 

However, the scenarios are more or less prone to the socio-economic divide. The strongest distinction in preferences among socio-economic segments was found in the case of ‘My Life in Between Realities’. This scenario is much more favoured by people under 35 years old, with the highest income (3rd tercile), and tertiary and upper secondary educated. In this scenario, society is organized individualistically and the driving sector is private. The spread of digital technologies and personalized services is the most important characteristic. On the other hand, the socioeconomic divide is smallest in case of preferences for the scenario ‘One for all, all for one’. In this scenario, the society is organized collectively, public sector dominates over private sector, and the key characteristic is localism. This appears to express to some extent a “digital divide” among the population. Population with more resources and educational skills are more prone to be benefited by the digitalization of the society, whereas older, less qualified, and lower socioeconomic population groups have more difficult accesses to the technologies and their potential benefits [53]. Results highlight the importance to address socioeconomic differences in access, skills and benefits of the digitalization of services in response to environmental challenge, by ensuring infrastructures, training and services adapted to the needs and resources across the social gradient.

### 4.3. What Value Orientations Would Explain Public Acceptability of the Healthier and More Environmentally Friendly Scenarios?

The importance of research on values lies in the ability of values to explain different beliefs and behaviours at the same time, as they are general in nature [54,55]. The healthier and more environmentally friendly scenarios are general visions of a future that include several policy measures and behaviours. For this reason, we think that values were able to explain the public acceptability of the scenarios. 

Three value orientations, specifically egoistic, altruistic, and biospheric were suggested as a theoretical basis of environmental concern and environment-related behaviours [28,29,30]. We empirically confirmed a distinction among the three value orientations. This finding adds to evidence previously found for other European countries [30]. We provide a new evidence that biospheric and egoistic value orientation is useful for examining the preferences for healthier and more environmentally friendly scenarios. 

Respondents with strong biospheric orientation tend to accept future scenarios. They particularly favour the ‘One for all, all for one’ scenario. The reason for this relationship might be the key characteristics, which are public sector and strong collectivism. People with egoistic orientations are most likely to select scenarios that place an emphasis on the role of the private sector in societal development, which are ‘My Life in Between Realities’ and ‘Our circular community’. These findings are in line with Schwartz’s Value Theory [27] and previous studies [30,31], as egoistic orientation motivates people to focus on their own personal interests (“self-enhancement” values), while biospheric orientation motivates people to transcend self-interest and accentuate impacts on nature (“self-transcendence” values). 

Additionally, our results support the Inclusion Model of Environmental Concerns (e.g., [56,57]), which admits that concerns about the environment include, progressively and cumulatively, egoistic, social and biospheric concerns. The model implies that both self-enhancement and self-transcendence value orientations can support pro-environmental options. More specifically, the model implies that people with stronger biospheric orientations are appealed by both self-enhancing and self-transcendent situation values, whereas people more oriented by egoistic values are mostly appealed by self-enhancing ones [56,57]. Results demonstrate the expected pattern: public acceptance for policy intervention increases with stronger biospheric concerns irrespective of the scenario, whereas, it increases with stronger egoist orientations in the scenarios perceived to be more protective of individuals self-interests, namely in which the role of the private sector and higher personalization of services are enhanced.

### 4.4. Limitations and Strengths

There are some limitations of this study. As we use cross-sectional data from questionnaire surveys, we cannot analyse actual referenda votes. We analyse choices of the scenarios in hypothetical situations. Thus, even when people are willing to support a scenario under specific conditions, we need to take into account that they might still fail to realize their intention in a real vote. Moreover, citizens may agree with the general policy principle, but may dislike the specific policy instruments and proposals meant to implement the principle (see [58]). To reduce this “principle-implementation gap”, the descriptions of the future of the domains presented to respondents included not only the desired outcome but the concrete measures to be taken to reach the outcomes as well. For example, the description of the transport system under the scenario ‘My Life in Between Realities’ contains information that public transport is highly interconnected and efficient and that price incentives are given for the use of public transport, biking and walking. In this scenario, there are fewer cars as it is more expensive to use them. 

Another limitation is the sample size, which doesn’t allow us to estimate multinomial logit models for each country and domain. Such models would be based on responses of about 160 persons. Instead, we control for effects of countries and domains in a multinomial logit model estimated for the dataset pooled for all countries and domains. 

Further, this study did not aim at letting respondents to choose among implementing measures in different domains. Due to the length of descriptions of the scenarios each respondent evaluated scenarios in one domain and not in all domains. The trade-offs of allocating governmental resources among domains might be focus of further research. On the other hand, an advantage of our approach is that domains can constitute experimental treatments, which allows us to analyse scenario choices under different contexts. 

Even though the future of the domains will be most likely a combination of different scenarios [59], it is important for policy makers to know what pathways people in their countries prefer and to be able to involve them in planning. Thus, the strengths of our study lie in examining the views of lay people on coherent scenarios of development of four domains of living in Europe in 2040. We ask them to choose the best concrete scenario instead of relying on an agreement with general goals. Our data comes from an original survey representative of adult populations (18 to 65 years) of five European countries. The potential support for the scenarios is explained by values and socio-economic characteristics. 

### 4.5. Policy Implications

Our findings indicate that the framing of the policy measures that would be aligned with the influential values and presented as a part of coherent scenario, could enhance public acceptability. While a previous literature review [60] on interventions to change health-related behaviours proposed to frame the interventions in a way that they would correspond to dominant core values, we show that the values that affect public acceptability of specific scenarios need to be first identified. In the case of our healthier and more environmentally friendly scenarios, the substantial potential to accept the governmental approaches seemed to be achieved by activating biospheric value orientation. Preference for the scenarios with a dominant role of the private sector was related also to an egoistic value orientation.

We can, therefore, infer that pro-environmental political interventions would resonate with a broader audience when policy messages and programs explicitly addressed selfish and biospheric concerns [56,57]. For example, to ensure broader public acceptance for a scenario like ‘One for all, all for one’, the potential gains for individual wealth must be enhanced (egoistic concerns), as well as the gains in the conservation of natural resources (biospheric concerns) within the scope of the localism represented in the scenario.

In a long term perspective, to support a gradual wider acceptance for sustainable life styles, we would suggest applying strategies to strengthen biospheric value orientation among people, as biospheric value orientation was found to be important for encouraging pro-environmental behaviours and public acceptability of environmental policies in several other studies [61]. Such strategies can include forming feelings of link between self and nature. This can be achieved through exposing people to nature, for example by promoting nature camps for children [62], by creating and improving parks and green spaces in urban areas [63], or anthropomorphizing nature [64]. Strengthening biospheric value orientation might be easier amongst children or adolescents who are still developing their value systems, but may also be feasible for adults. Even though values are quite stable during a lifetime, they may change, when adults are motivated repetitively to reassess their value system [61,65].

To enhance the public support for the policies, when they are actually implemented, it is important to enable people to act on their values by various strategies [61], such as changing the costs and benefits of behaviour using pricing instruments, raising awareness using information and marketing campaigns, changing facilitating conditions and situational factors (access to healthy and sustainable food, public transport etc.), changing institutional and cultural context, helping communities to help themselves [66]. These strategies, particularly changing the costs and benefits of behaviour, can make egoistic values compatible with biospheric values [67]. 

## 5. Conclusions

Implementation of policies that would lead to an improvement of health and the environment might be feasible in five European countries. We find that a majority of people in different countries (ranging from 74% to 93%, depending on the country) would accept one of the healthier and more environmentally friendly scenarios according to our results based on a representative questionnaire survey. Although the public acceptability seems to be large, it differs significantly according to socioeconomic segments and the underlying values of different people. Overall, people aged over 35 years old, those who are less educated, and those in the lowest household income tertile were less supportive of all scenarios than the others. These socioeconomic differences in preferences were stronger in the case of the scenarios in which the society is organized individualistically and is driven by private sector with emphasis on digital technologies and personalized services. Smaller socioeconomic distinctions were found for scenarios in which the society is organized collectively and public sector, particularly local authorities, are the driving forces of living. People with strong biospheric value orientations are more likely to accept all future scenarios except the individualistically organized and private sector driven scenario. They particularly prefer the scenarios driven by the public sector and strong collectivism. The likelihood of choosing scenarios with a dominant role for the private sector was related to having an egoistic value orientation. 

To our knowledge, this is the first study that has examined the preferences of the public for future scenarios that would encourage “triple win” solutions (healthier, more sustainable lifestyles, as well as greater health equity) in four domains of living (green spaces, active mobility, energy efficient housing and food consumption). These results imply that there may be important actions to be taken to help build consensus on policy actions to promote the “triple win”—with the need to consider the preferences of people with different value systems in the design and communication of policy and potentially for action to promote certain value systems through education or other actions. Inaction is clearly not the preferred choice—people do have preferences for change and reconciling differences in these preferences in the formation of policy will be challenging. To bring about real change and the “triple win” across different sectors requires a better understanding of what futures people want. It is our hope that this study gives a deeper insight into the drivers behind those preferences and may help in a small way to us realising a healthier, more sustainable future with less health inequalities. 

## Figures and Tables

**Table 1:** Definition of the future scenarios ^1^.

Scenario	Driving Sector	Social Processes	Description
My life between realities	private sector	individualistic	The main vision is the interconnectivity—everything is digital, connected and personalized. A better future is supported by big data which drive efficiency and performance and enable high-quality and personalized products and services. Development is driven by business and complemented by a governmental intervention.
Less is more to me	public sector	individualistic	Material ownership is less important and the influence of the private sector declines. The government also ensures the provision and management of products and services (including access to health care services and education). There is a tendency towards greater sufficiency.
One for all, all for one	public sector	collectivist	The main characteristic is the localism which is reflected in the life of society (local diets, community interactions, leisure activities etc.). Support for this future is formed by technological innovations and rules and regulations of policy makers.
Our circular community	private sector	collectivist	Society is highly connected and dependent on technology. More emphasis is on commonly-owned and created goods and more efficient services and products. Economy is a closed-loop and service is more important than product ownership.

^1^ Sources: [11,14].

**Table 2:** Support for healthier, more equitable and more sustainable future scenarios—multinomial logit model, adjusted odds ratios ^1^.

Scenario	My Life in between Realities	Less Is More to Me	One for All, All for One	Our Circular Community
(Intercept)	0.21 (0.11–0.42) ***	0.19 (0.1–0.37) ***	0.61 (0.32–1.15)	0.16 (0.08–0.31) ***
Domain (food-reference)				
Green spaces	0.8 (0.51–1.24)	1.68 (1.2–2.36) **	1.15 (0.83–1.58)	5.83 (4.12–8.26) ***
Active mobility	2.16 (1.56–2.99) ***	1 (0.74–1.36)	0.42 (0.31–0.56) ***	1.38 (0.99–1.94)
Housing	1.9 (1.35–2.68) ***	0.99 (0.72–1.36)	0.46 (0.34–0.64) ***	3.03 (2.18–4.21) ***
Country (CZ-reference)				
UK	0.86 (0.58–1.26)	1.03 (0.72–1.47)	0.86 (0.6–1.22)	0.82 (0.58–1.17)
LV	1.36 (0.9–2.06)	1.45 (0.98–2.14)	1.21 (0.82–1.79)	1.08 (0.73–1.59)
ES	1.22 (0.79–1.88)	1.31 (0.87–1.97)	0.91 (0.61–1.37)	1.6 (1.08–2.36) *
PT	1.21 (0.77–1.89)	0.88 (0.57–1.35)	0.9 (0.59–1.36)	1.34 (0.89–2)
Gender (female-reference)				
Male	1.23 (0.96–1.58)	1.03 (0.81–1.3)	1.05 (0.83–1.32)	1.2 (0.96–1.51)
Age (18–34 years-reference)				
35–49 years	0.48 (0.35–0.65) ***	0.46 (0.35–0.62) ***	0.62 (0.46–0.83) **	0.56 (0.42–0.74) ***
50–65 years	0.47 (0.34–0.66) ***	0.52 (0.38–0.72) ***	0.72 (0.53–0.99) *	0.44 (0.32–0.61) ***
Household income (1st tercile reference)				
2nd tercile	1.25 (0.9–1.75)	1.45 (1.06–1.98) *	1.32 (0.98–1.79)	1.52 (1.12–2.05) **
3rd tercile	1.73 (1.22–2.46) **	1.65 (1.18–2.3) **	1.44 (1.04–2) *	1.55 (1.12–2.15) **
missing	0.61 (0.41–0.89) *	0.58 (0.4–0.82) **	0.46 (0.32–0.65) ***	0.59 (0.42–0.84) **
Municipality size (up to 4999 people-reference)				
5000 or more	1.27 (0.96–1.68)	1.24 (0.95–1.61)	0.98 (0.76–1.26)	1.2 (0.93–1.55)
Education (primary and lower secondary-reference)				
upper secondary	1.58 (1.16–2.16) **	1.56 (1.16–2.09) **	1.45 (1.09–1.92) *	1.39 (1.06–1.84) *
tertiary	1.93 (1.39–2.69) ***	1.82 (1.33–2.5) ***	1.41 (1.03–1.93) *	1.49 (1.1–2.02) **
Chronic disease	0.99 (0.76–1.31)	1.14 (0.88–1.46)	0.94 (0.73–1.21)	0.93 (0.73–1.2)
Values				
altruistic	1.07 (0.95–1.21)	1.02 (0.91–1.14)	1.09 (0.97–1.22)	1.03 (0.92–1.16)
biospheric	1.12 (1.01–1.24) *	1.17 (1.06–1.29) **	1.2 (1.09–1.33) ***	1.15 (1.05–1.27) **
egoistic	1.14 (1.05–1.25) **	1.07 (0.99–1.16)	0.99 (0.92–1.07)	1.1 (1.02–1.19) *
hedonism	0.94 (0.84–1.05)	0.98 (0.89–1.09)	0.97 (0.88–1.08)	0.94 (0.85–1.04)
security	0.95 (0.84–1.08)	1.09 (0.97–1.23)	1 (0.88–1.12)	1.06 (0.94–1.19)
log Lik.’	−5020.748			
AIC	10225.5			
N	*3222*			

^1^ Note: Data are presented as adjusted odds ratio (OR), 95% confidence interval around OR reported in brackets. Significance levels: * *p* < 0.05; ** *p* < 0.01; *** *p* < 0.001.

## Supplementary Materials

The following are available online at https://www.mdpi.com/1660-4601/17/17/6071/s1, Table S1: Proportion of people according to sociodemographic characteristics in national populations aged, Table S2: Proportion of people according to region in national populations aged between 18 and 65, Table S3: Percentages of scenario choices by the survey variables used in the multinomial logit models, Figure S1: Boxplots of value variables by scenario choices, Table S4. Pearson Chi-Square tests for associations between countries and choices of scenarios for different domains, Table S5. Post-hoc analysis after chi-square tests shown in Table S4, with Bonferroni adjustment, Table S6a. Multinomial logit model with “None/Don’t know” as a reference category different reference categories—adjusted odds ratio (OR) (95% confidence interval around OR), Table S6b. Multinomial logit model with “Our circular community” as a reference category—adjusted odds ratio (OR) (95% confidence interval around OR), Table S6c. Multinomial logit model with “One for all, all for one” as a reference category—adjusted odds ratio (OR) (95% confidence interval around OR), Table S6d. Multinomial logit model with “Less is more to me” as a reference category—adjusted odds ratio (OR) (95% confidence interval around OR), Table S6e. Multinomial logit model with “My Life in Between Realities” as a reference category—adjusted odds ratio (OR) (95% confidence interval around OR).

## Appendix A

**Table A1:** Items to measure values and reliabilities of the value constructs (Cronbach’s α) ^1^.

Value Item	Cronbach’s α
*Altruistic value orientation*	0.82
A world of peace (free of war and conflicts)	
Equality (equal opportunity for all)	
Social justice (righting injustice, care for the weak)	
*Biospheric value orientation*	0.9
Preventing environmental pollution (protection of natural resources)	
Protecting the environment (preserving nature)	
Respecting the earth (harmony with other species)	
*Egoistic value orientation*	0.73
Authority (the right to lead or command)	
Influence (having an impact on people and events)	
Wealth (material possessions, money)	
*Hedonism*	0.8
Pleasure (joy, gratification of desires)	
Enjoying life (enjoying food, sex, leisure etc.)	
Self-indulgence (doing pleasant things)	
*Security*	0.73
Healthy (not being sick physically or mentally)	
Reciprocation of favours (avoidance of indebtedness)	
Family security (safety for loved ones)	

^1^ Wording of items adopted from Schwartz’s Value Theory [27]. We followed typology of altruistic, biospheric and egoistic value orientations [28,29,30].
