# Supplementary material for: How to Achieve a Healthier and More Sustainable Europe by 2040 According to the Public? Results of a Five-Country Questionnaire Survey
Source: Int J Environ Res Public Health. 2020 Aug 20;17(17):6071. doi: 10.3390/ijerph17176071 (PMC7503986; doi:10.3390/ijerph17176071)
Supplement: Supplementary file 1 [file ijerph-17-06071-s001.pdf]

Supplementary Material

Table S1. Proportion of people according to sociodemographic characteristics in national populations aged between 18 and 65[1].

	CZ	UK	LV	ES	PT
Gender					
Male	51%	50%	49%	50%	48%
Female	49%	50%	51%	50%	52%
Age					
18–34 y.o.	32%	36,2%	33%	29%	30%
35–50 y.o.	38%	34%	34%	41%	38%
51–69 y.o.	30%	30%	33%	30%	32%
Education					
primary and lower secondary	43%	19%	11%	40%	49%
upper secondary	37%	41%	57%	26%	28%
tertiary	20%	40%	31%	34%	23%

[1]Sources for setting the national quotas: EUROSTAT, data for year 2017, https://ec.europa.eu/eurostat

Table S2. Proportion of people according to region in national populations aged between 18 and 65[1].

CZ		UK		ES		PT	
**Prague**	**13%**	**North East**	**4%**	Noroeste	9%	Norte	35%
Central Bohemia	12%	North West	11%	Noreste	9%	Algarve	4%
South Bohemia	6%	Yorkshire and the Humber	8%	Comunidad De Madrid	14%	Centro	22%
Plzeňský	5%	East Midlands	7%	Centro	12%	Área Metropolitana de Lisboa	27%
Karlovarský	3%	West Midlands	9%	Este	29%	Alentejo	7%
Ústecký	8%	East of England	9%	Sur	21%	Região Autónoma dos Açores	2%
Liberecký	4%	Greater London	14%	Canarias	5%	Região Autónoma da Madeira	3%
Královéhradecký	5%	South East (excluding London)	13%				
Pardubický	5%	South West	8%				
Vysočina	5%	Wales	5%				
South Moravia	11%	Scotland	9%				
Olomoucký	6%	Northern Ireland	3%				
Zlínský	5%						
Moravia-Silesia	12%						

[1]*Quota for Latvian regions was not set.*

Table S3. Percentages of scenario choices by the survey variables used in the multinomial logit models.

Scenario	Our circular community	One for all, all for one	Less is more to me	My Life in Between Realities	I don't know/ None	Total
Domain						
Green spaces	38%	26%	24%	8%	16%	24%
Active mobility	18%	19%	28%	43%	31%	27%
Housing	33%	17%	23%	31%	26%	26%
Food	12%	39%	25%	18%	27%	24%
Country						
UK	25%	30%	31%	29%	32%	29%
CZ	17%	20%	17%	16%	20%	18%
LV	16%	19%	21%	21%	17%	19%
ES	23%	16%	19%	18%	16%	19%
PT	18%	15%	12%	16%	15%	15%
Gender						
Female	49%	52%	53%	47%	51%	50%
Male	51%	48%	47%	53%	49%	50%
Age						
18–34 years	34%	30%	36%	39%	24%	32%
35–49 years	40%	35%	32%	34%	42%	37%
50–65 years	26%	35%	32%	27%	35%	31%
Household income						
1st tercile	22%	26%	21%	21%	28%	23%
2nd tercile	33%	32%	32%	29%	27%	31%
3rd tercile	32%	30%	33%	36%	22%	31%
missing	13%	12%	13%	14%	24%	15%
Municipality size						
up to 4,999 people	24%	29%	24%	23%	29%	26%
5,000 or more	76%	71%	76%	77%	71%	74%
Education						
primary and lower secondary	31%	31%	25%	25%	40%	30%
upper secondary	38%	41%	40%	38%	35%	38%
tertiary	31%	28%	35%	36%	25%	31%
Chronic disease						
None	76%	69%	65%	58%	72%	69%
Chronic disease	24%	31%	35%	42%	28%	31%

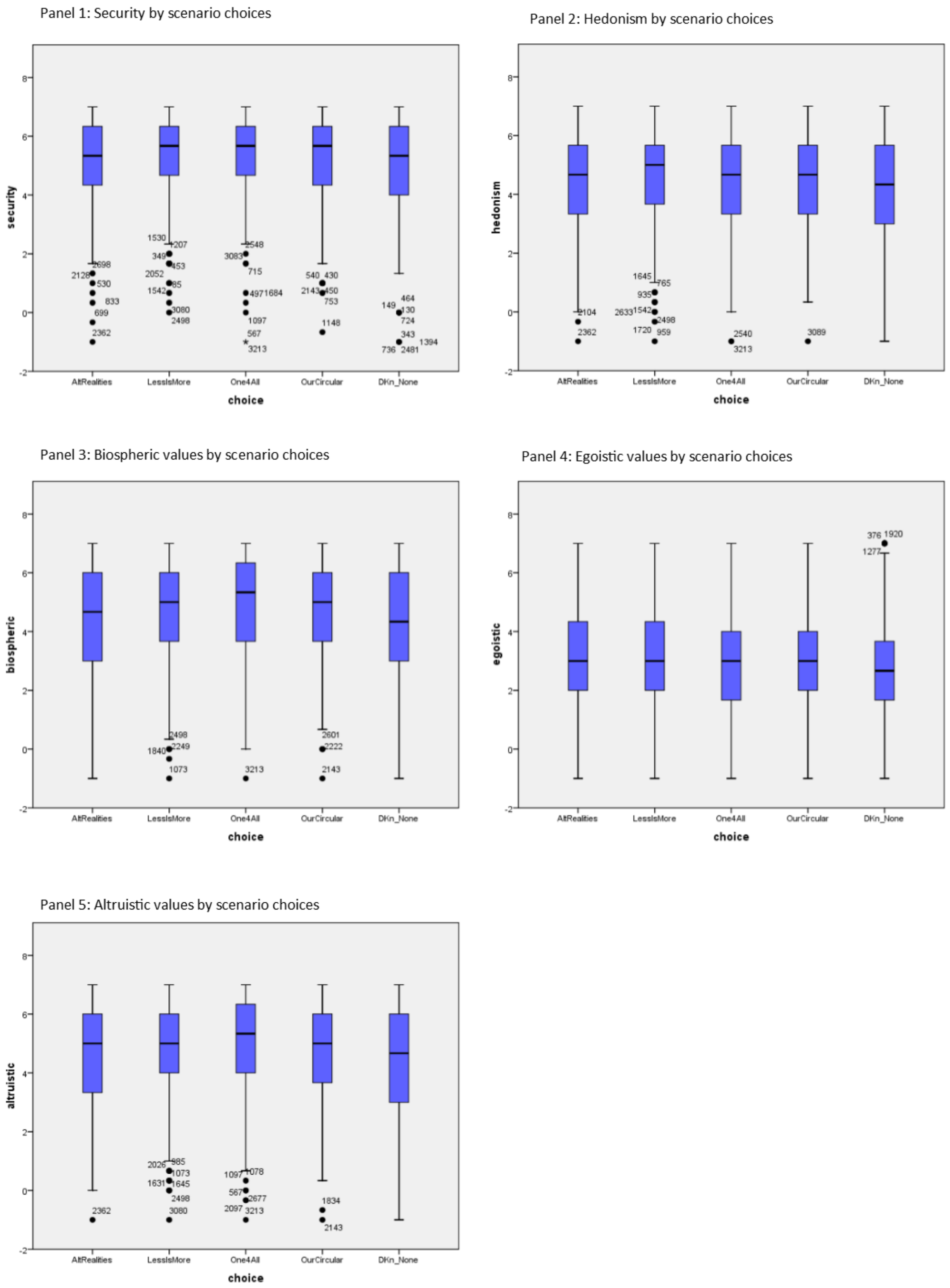

Figure 1. Boxplots of value variables by scenario choices.

Table S4. Pearson Chi-Square tests for associations between countries and choices of scenarios for different domains.

	**Value**	**df**	**Asymp. Sig. (2-sided)**
Active mobility	35.424	16	0.003
Energy efficient housing	57.068	16	0.000
Food consumption	24.201	16	0.085

Green spaces	22.087	16	0.140

Table S5. Post-hoc analysis after chi-square tests shown in Table S4, with Bonferroni adjustment.

		Scenario	My Life in Between Realities	Less is more to me	One for all, all for one	Our circular community	None/ Don't know
Mobility	CZ	Adjusted Residual	−2.6	−0.8	1.7	1.6	0.7
		p values	0.008	0.433	0.091	0.101	0.509
	ES	Adjusted Residual	1.1	−1.0	−0.8	2.3	−1.5
		p values	0.288	0.303	0.410	0.023	0.140
	LV	Adjusted Residual	1.1	0.5	−1.0	−0.3	−0.6
		p values	0.251	0.645	0.311	0.772	0.577
	PT	Adjusted Residual	1.0	−1.9	0.2	1.0	−0.2
		p values	0.327	0.054	0.847	0.341	0.878
	UK	Adjusted Residual	−0.4	2.7	0.0	−3.9	1.3
		p values	0.679	0.007	0.988	0.000*	0.184
Housing	CZ	Adjusted Residual	0.8	−0.3	−0.2	−1.9	2.0
		p values	0.453	0.776	0.807	0.064	0.046
	ES	Adjusted Residual	−0.6	−0.2	−2.4	4.4	−2.5
		p values	0.581	0.858	0.017	0.000*	0.013
	LV	Adjusted Residual	0.9	1.7	3.0	−3.2	−1.4
		p values	0.374	0.086	0.003	0.001*	0.155
	PT	Adjusted Residual	−0.5	−1.5	−2.2	3.2	0.1
		p values	0.588	0.144	0.027	0.001*	0.890
	UK	Adjusted Residual	−0.4	0.2	1.6	−2.2	1.6
		p values	0.659	0.878	0.108	0.027	0.119

[1]Significance level: * p <0.002 (threshold after Bonferroni adjustment)

Table S5. *Cont.* Post-hoc analysis after chi-square tests shown in Table S4, with Bonferroni adjustment.

		Scenario	My Life in Between Realities	Less is more to me	One for all, all for one	Our circular communi ty	None/ Don't know
Food	CZ	Adjusted Residual	−0.7	−0.6	0.6	0.8	−0.2
		p values	0.511	0.536	0.521	0.428	0.813
	ES	Adjusted Residual	−1.0	3.1	−2.3	0.6	−0.1
		p values	0.296	0.0021	0.022	0.524	0.924
	LV	Count	21	30	54	6	30
		Adjusted Residual	1.1	0.0	1.1	−3.2	0.4
		p values	0.270	0.968	0.254	0.002*	0.713
	PT	Adjusted Residual	0.5	−0.5	−0.1	−0.4	0.6
		p values	0.609	0.584	0.947	0.686	0.581
	UK	Adjusted Residual	0.1	−1.6	0.5	1.7	−0.4
		p values	0.920	0.111	0.614	0.086	0.653
Green spaces	CZ	Adjusted Residual	1.1	−0.5	1.3	−1.4	0.1
		p values	0.265	0.640	0.178	0.169	0.891
	ES	Adjusted Residual	−1.1	−1.5	1.3	0.1	0.7
		p values	0.258	0.140	0.182	0.897	0.488
	LV	Adjusted Residual	−0.9	2.0	−2.1	1.0	−0.7
		p values	0.379	0.047	0.037	0.310	0.490
	PT	Adjusted Residual	−0.7	−1.1	1.1	1.3	−1.5
		p values	0.462	0.287	0.282	0.189	0.126
	UK	Adjusted Residual	1.4	0.8	−1.4	−0.9	1.1
		p values	0.176	0.428	0.172	0.392	0.261

[1]Significance level: * p <0.002 (threshold after Bonferroni adjustment)

Table S6a. Multinomial logit model with "None/Don't know" as a reference category - adjusted odds ratio (OR) (95% confidence interval around OR).

Scenario	My Life in Between Realities	Less is more to me	One for all, all for one	Our circular community
(Intercept)	0.278 (0.156–0.496) ***	0.228 (0.129–0.401) ***	0.588 (0.341–1.015)	0.193 (0.111–0.334) ***
Domain (food - reference)				
Green spaces	0.79 (0.51–1.226)	1.661 (1.188–2.321) **	1.134 (0.825–1.558)	5.728 (4.065–8.071) ***
Mobility	2.177 (1.581–2.997) ***	1.011 (0.751–1.363)	0.421 (0.312–0.568) ***	1.4 (1.003–1.954) *
Housing	1.946 (1.391–2.723) ***	1.005 (0.735–1.374)	0.476 (0.349–0.649) ***	3.07 (2.222–4.242) ***
Country (CZ - reference)				
UK	1.018 (0.707–1.468)	1.191 (0.846–1.676)	0.938 (0.672–1.311)	0.916 (0.655–1.281)
LV	1.46 (0.984–2.167)	1.561 (1.076–2.263) *	1.243 (0.859–1.798)	1.093 (0.755–1.584)
ES	1.233 (0.813–1.872)	1.326 (0.895–1.965)	0.899 (0.607–1.333)	1.616 (1.109–2.355) *
PT	1.049 (0.68–1.618)	0.767 (0.503–1.17)	0.791 (0.526–1.187)	1.186 (0.802–1.754)
Values				
altruistic	1.027 (0.913–1.155)	0.991 (0.887–1.106)	1.056 (0.946–1.179)	0.992 (0.89–1.105)
biospheric	1.091 (0.984–1.209)	1.147 (1.041–1.263) **	1.189 (1.079–1.311) ***	1.131 (1.028–1.245) *
egoistic	1.173 (1.079–1.277) ***	1.089 (1.008–1.178) *	1 (0.925–1.08)	1.125 (1.042–1.215) **
hedonism	1.012 (0.911–1.125)	1.042 (0.944–1.151)	1.011 (0.917–1.115)	1.007 (0.914–1.11)
security	0.92 (0.814–1.041)	1.067 (0.948–1.2)	0.993 (0.884–1.116)	1.019 (0.908–1.143)

[1]Significance levels: * p <0.05; ** p <0.01; *** p <0.001.

Table S6b. Multinomial logit model with "Our circular community" as a reference category - adjusted odds ratio (OR) (95% confidence interval around OR).

Scenario	My Life in Between Realities	None/Don't know	Less is more to me	One for all, all for one
(Intercept)	1.444 (0.798–2.611)	5.194 (2.99–9.022) ***	1.182 (0.67–2.087)	3.055 (1.754–5.319) ***
Domain (food - reference)				
Green spaces	0.138 (0.09–0.211) ***	0.175 (0.124–0.246) ***	0.29 (0.212–0.397) ***	0.198 (0.147–0.267) ***
Mobility	1.555 (1.096–2.206) *	0.714 (0.512–0.997) *	0.723 (0.52–1.004)	0.301 (0.216–0.419) ***
Housing	0.634 (0.45–0.892) **	0.326 (0.236–0.45) ***	0.327 (0.238–0.45) ***	0.155 (0.113–0.213) ***
Country (CZ - reference)				
UK	1.112 (0.774–1.598)	1.092 (0.781–1.527)	1.3 (0.933–1.812)	1.025 (0.74–1.419)
LV	1.336 (0.91–1.961)	0.915 (0.631–1.325)	1.428 (1.004–2.03) *	1.137 (0.801–1.615)
ES	0.763 (0.515–1.131)	0.619 (0.425–0.901) *	0.82 (0.572–1.176)	0.556 (0.387–0.8) **
PT	0.885 (0.586–1.336)	0.843 (0.57–1.247)	0.647 (0.437–0.958) *	0.667 (0.457–0.973) *
Values				
altruistic	1.036 (0.924–1.162)	1.009 (0.905–1.124)	0.999 (0.899–1.11)	1.065 (0.957–1.185)
biospheric	0.964 (0.871–1.068)	0.884 (0.803–0.973) *	1.014 (0.923–1.114)	1.051 (0.956–1.156)
egoistic	1.043 (0.963–1.129)	0.889 (0.823–0.959) **	0.968 (0.901–1.04)	0.888 (0.827–0.954) **
hedonism	1.005 (0.907–1.114)	0.993 (0.901–1.094)	1.035 (0.941–1.138)	1.004 (0.914–1.103)
security	0.904 (0.8–1.021)	0.982 (0.875–1.101)	1.047 (0.933–1.175)	0.975 (0.869–1.093)

[1] Significance levels: * p <0.05; ** p <0.01; *** p <0.001.

Table S6c. Multinomial logit model with "One for all, all for one" as a reference category - adjusted odds ratio (OR) (95% confidence interval around OR).

Scenario	My Life in Between Realities	None/Don't know	Less is more to me	Our circular community
(Intercept)	0.473 (0.261–0.856) *	1.7 (0.985–2.936)	0.387 (0.22–0.683) **	0.327 (0.188–0.57) ***
Domain (food - reference)				
Green spaces	0.697 (0.465–1.045)	0.882 (0.642–1.212)	1.465 (1.099–1.952) **	5.052 (3.752–6.802) ***
Mobility	5.169 (3.76–7.106) ***	2.374 (1.76–3.203) ***	2.402 (1.789–3.225) ***	3.324 (2.386–4.63) ***
Housing	4.091 (2.938–5.696) ***	2.102 (1.541–2.868) ***	2.113 (1.556–2.869) ***	6.453 (4.697–8.865) ***
Country (CZ - reference)				
UK	1.085 (0.754–1.563)	1.066 (0.763–1.489)	1.269 (0.91–1.769)	0.976 (0.705–1.352)
LV	1.175 (0.799–1.727)	0.804 (0.556–1.163)	1.256 (0.883–1.785)	0.879 (0.619–1.249)
ES	1.371 (0.907–2.074)	1.112 (0.75–1.648)	1.474 (1.009–2.155) *	1.797 (1.25–2.583) **
PT	1.327 (0.863–2.041)	1.265 (0.842–1.9)	0.97 (0.644–1.461)	1.5 (1.028–2.189) *
Values				
altruistic	0.973 (0.864–1.095)	0.947 (0.848–1.057)	0.938 (0.842–1.045)	0.939 (0.844–1.045)
biospheric	0.917 (0.826–1.018)	0.841 (0.763–0.927) ***	0.964 (0.876–1.062)	0.951 (0.865–1.046)
egoistic	1.174 (1.082–1.273) ***	1 (0.926–1.081)	1.09 (1.013–1.172) *	1.126 (1.048–1.209) **
hedonism	1.001 (0.902–1.111)	0.989 (0.897–1.09)	1.03 (0.937–1.133)	0.996 (0.907–1.094)
security	0.927 (0.818–1.05)	1.007 (0.896–1.132)	1.074 (0.955–1.208)	1.026 (0.915–1.15)

[1]Significance levels: * p <0.05; ** p <0.01; *** p <0.001.

Table S6d. Multinomial logit model with "Less is more to me" as a reference category - adjusted odds ratio (OR) (95% confidence interval around OR).

Scenario	My Life in Between Realities	None/Don't know	One for all, all for one	Our circular community
(Intercept)	1.221 (0.668–2.23)	4.393 (2.495–7.734) ***	2.583 (1.465–4.555) **	0.846 (0.479–1.492)
Domain (food - reference)				
Green spaces	0.476 (0.313–0.723) ***	0.602 (0.431–0.841) **	0.683 (0.512–0.91) **	3.449 (2.519–4.723) ***
Mobility	2.152 (1.57–2.951) ***	0.989 (0.734–1.332)	0.416 (0.31–0.559) ***	1.384 (0.996–1.924)
Housing	1.936 (1.39–2.698) ***	0.995 (0.728–1.36)	0.473 (0.349–0.643) ***	3.054 (2.221–4.2) ***
Country (CZ - reference)				
UK	0.855 (0.592–1.235)	0.84 (0.597–1.182)	0.788 (0.565–1.098)	0.769 (0.552–1.072)
LV	0.935 (0.638–1.372)	0.641 (0.442–0.929) *	0.796 (0.56–1.132)	0.7 (0.493–0.996) *
ES	0.93 (0.618–1.399)	0.754 (0.509–1.118)	0.678 (0.464–0.991) *	1.219 (0.85–1.748)
PT	1.368 (0.881–2.125)	1.304 (0.855–1.99)	1.031 (0.685–1.552)	1.546 (1.044–2.29) *
Values				
altruistic	1.037 (0.923–1.165)	1.009 (0.904–1.127)	1.066 (0.957–1.188)	1.001 (0.9–1.113)
biospheric	0.951 (0.858–1.054)	0.872 (0.791–0.961) **	1.037 (0.942–1.142)	0.986 (0.898–1.084)
egoistic	1.077 (0.994–1.168)	0.918 (0.849–0.992) *	0.918 (0.853–0.987) *	1.033 (0.962–1.109)
hedonism	0.972 (0.875–1.078)	0.96 (0.869–1.059)	0.97 (0.882–1.068)	0.966 (0.879–1.063)
security	0.863 (0.762–0.977) *	0.937 (0.833–1.055)	0.931 (0.828–1.047)	0.955 (0.851–1.072)

[1]Significance levels: * p <0.05; ** p <0.01; *** p <0.001.

Table S6e. Multinomial logit model with "My Life in Between Realities" as a reference category - adjusted odds ratio (OR) (95% confidence interval around OR).

Scenario	None/Don't know	Less is more to me	One for all, all for one	Our circular community
(Intercept)	3.598 (2.016–6.423) ***	0.819 (0.448–1.496)	2.116 (1.169–3.831) *	0.693 (0.383–1.253)
Domain (food - reference)				
Green spaces	1.265 (0.816–1.963)	2.101 (1.384–3.191) ***	1.435 (0.957–2.151)	7.247 (4.742–11.077) ***
Mobility	0.459 (0.334–0.632) ***	0.465 (0.339–0.637) ***	0.193 (0.141–0.266) ***	0.643 (0.453–0.912) *
Housing	0.514 (0.367–0.719) ***	0.516 (0.371–0.72) ***	0.244 (0.176–0.34) ***	1.578 (1.121–2.22) **
Country (CZ - reference)				
UK	0.982 (0.681–1.415)	1.169 (0.81–1.688)	0.921 (0.64–1.327)	0.899 (0.626–1.292)
LV	0.685 (0.461–1.016)	1.069 (0.729–1.568)	0.851 (0.579–1.252)	0.749 (0.51–1.099)
ES	0.811 (0.534–1.23)	1.075 (0.715–1.618)	0.729 (0.482–1.103)	1.311 (0.884–1.942)
PT	0.953 (0.618–1.47)	0.731 (0.471–1.135)	0.753 (0.49–1.159)	1.13 (0.748–1.707)
Values				
altruistic	0.973 (0.866–1.095)	0.964 (0.858–1.084)	1.028 (0.913–1.157)	0.965 (0.86–1.083)
biospheric	0.917 (0.827–1.016)	1.051 (0.949–1.165)	1.09 (0.982–1.21)	1.037 (0.937–1.148)
egoistic	0.852 (0.783–0.927) ***	0.928 (0.856–1.006)	0.852 (0.785–0.924) ***	0.959 (0.886–1.038)
hedonism	0.988 (0.889–1.097)	1.029 (0.927–1.142)	0.999 (0.9–1.109)	0.995 (0.897–1.103)
security	1.086 (0.961–1.228)	1.159 (1.023–1.313) *	1.079 (0.952–1.222)	1.107 (0.98–1.25)

[1]Significance levels: * p <0.05; ** p <0.01; *** p <0.001.
